# Association between serum vitamin B_6_ levels and depression in adults: a cross-sectional National Health and Nutrition Examination Survey (NHANES) study

**DOI:** 10.1017/S0007114526106321

**Published:** 2026-05-14

**Authors:** Jiangxu Mai, Yanqing Wang, Jingxin Li, Xiuli Zuo

**Affiliations:** 1 Shandong Provincial Clinical Research Center for Digestive Disease, Qilu Hospital of Shandong University, People’s Republic of China; 2 Department of Gastroenterology, Qilu Hospital of Shandong University, People’s Republic of China; 3 Department of Physiology, School of Basic Medical Sciences, Cheeloo College of Medicine, People’s Republic of China; 4 Department of Neuroscience and Friedman Brain Institute, Icahn School of Medicine at Mount Sinai, New York, NY, USA

**Keywords:** Vitamin B_6_, depression, pyridoxal 5′-phosphate, 4-pyridoxal acid, National Health and Nutrition Examination Survey

## Abstract

Vitamin B_6_ is implicated in multiple mental disorders, and accumulating evidence suggests an inverse relationship with depression; however, important aspects of the underlying dose–response patterns and the roles of individual circulating vitamin B_6_ metabolites remain incompletely understood. We analysed data from the National Health and Nutrition Examination Survey 2005–2010. Depression was defined as a Patient Health Questionnaire-9 score ≥10. Vitamin B_6_ status was assessed using serum pyridoxal 5′-phosphate (PLP), the biologically active coenzyme form, and 4-pyridoxic acid (PA), the principal catabolic and urinary excretion product of vitamin B_6_. Among 12 620 participants, 1070 (8·5 %) met criteria for depression. After adjusting for relevant covariates, multiple logistic regression revealed that individuals in higher quartiles of serum PLP and PA (Q2–Q4) had significantly lower odds of depression compared with those in the lowest quartile (Q1). Restricted cubic spline analyses identified nonlinear relationships: L-shaped for PLP (P-nonlinearity = 0·001) and U-shaped for PA (P-nonlinearity = 0·017). Below the inflection points (90·7 nmol/L for PLP; 73·9 nmol/L for PA), both metabolites showed significant inverse associations with depression (PLP: OR = 0·992, 95 % CI: 0·988–0·996, *P* < 0·001; PA: OR = 0·994, 95 % CI: 0·993–0·996, *P* < 0·001). Above these thresholds, the association became non-significant for PLP (*P* = 0·353), while PA demonstrated a positive association with depression (OR = 1·008, 95 % CI: 1·002–1·013, *P* < 0·01). Subgroup analyses confirmed the robustness of these inverse associations across demographic categories. Serum vitamin B_6_ metabolites, PLP and its excretion product PA, exhibit non-linear associations with depression, with distinct threshold effects and metabolite-specific patterns that likely reflect both vitamin B_6_ availability and turnover.

Depression is a prevalent and debilitating affective disorder characterised by persistent sadness and anhedonia^(1)^. As a leading risk factor for suicide, depression is recognised globally as a significant impediment to both mental and physical well-being^(2)^. According to recent WHO estimates, depression affects approximately 5 % of adults worldwide, with a disproportionate impact on women^(3,4)^. The COVID-19 pandemic has further exacerbated this burden, with an estimated 27·6 % increase in major depressive disorder cases globally^(3)^. Given depression’s substantial contribution to the global burden of disease, investigating both risk factors and preventive strategies remains imperative.

While pharmacological interventions remain the mainstay of treatment, conventional antidepressants are frequently associated with adverse effects that limit adherence and efficacy^(5)^. Consequently, research interest has increasingly focused on the potential role of nutritional factors in both the pathogenesis and management of depressive disorders^(6,7)^. Vitamin B_6_, an essential water-soluble nutrient, comprises a family of six compounds, with pyridoxal-5-phosphate (PLP) representing the predominant biologically active form in plasma^(8)^. PLP functions as a critical cofactor in numerous enzymatic reactions implicated in depression pathophysiology, including the clearance of reactive oxygen species, homocysteine metabolism and the synthesis of neurotransmitters such as serotonin, dopamine and γ-aminobutyric acid (GABA)^(9)^. 4-Pyridoxic acid (PA), the principal catabolite of vitamin B_6_, is readily detectable in plasma and has been extensively validated as a reliable biomarker for comprehensive assessment of vitamin B_6_ status in clinical settings^(10)^. The measurement of PA, particularly in conjunction with PLP levels, provides valuable insights into vitamin B_6_ metabolism and turnover, which may have implications for understanding its role in neuropsychiatric conditions.

However, studies examining the relationship between vitamin B_6_ and depression have yielded inconsistent findings. A cross-sectional cohort study of community-dwelling older adults in Ireland demonstrated that individuals in the lowest quintile of serum PLP had elevated depression risk^(11)^. Research among Iranian adults aged 18–69 years revealed that increased vitamin B_6_ intake correlated with reduced depression risk in women, but this association was absent in men^(12)^. An intervention study reported that patients with depression had significantly lower plasma PLP concentrations but higher PA-based functional indices of vitamin B_6_ status compared with healthy controls^(13)^. This pattern suggests increased utilisation and catabolism of vitamin B_6_ in depressive states, with PLP depletion accompanied by enhanced conversion to the excretion product PA. Conversely, an investigation of Hispanic elderly participants found no significant correlation between serum PLP and depression^(14)^. These contradictory findings may stem from methodological differences, including variations in study populations, assessment tools for depression, analytical techniques for vitamin B_6_ metabolites and insufficient adjustment for confounding factors. Additionally, most previous research has focused on older populations and clinical cohorts rather than representative samples of the general adult population. Recently, Lu et al. reported that higher dietary vitamin B_6_ intake and higher plasma PLP quartiles were associated with a lower prevalence of depression in USA adults, with an approximately linear inverse dose–response relationship^(15)^. Their study provides important population-based evidence linking vitamin B_6_ status to depression risk. However, that analysis did not evaluate circulating 4-pyridoxic acid (PA), a key catabolite of vitamin B_6_, nor did it characterise potential non-linear threshold effects for vitamin B_6_ metabolites. In addition to observational evidence, interventional data also support a potential causal role of vitamin B_6_ in mood regulation. A recent double-blind randomised controlled trial in young adults reported that 1 month of high-dose vitamin B_6_ supplementation (100 mg/day) reduced self-reported anxiety and showed a trend towards reduced depressive symptoms compared with placebo, consistent with a role of vitamin B_6_ in enhancing GABAergic inhibition^(16)^. Consequently, the broader pattern of vitamin B_6_ metabolism in relation to depression risk, particularly the role of PA, remains to be clarified.

Therefore, we aimed to investigate the association between serum PLP, PA and depression risk in a nationally representative sample of USA adults using The National Health and Nutrition Examination Survey (NHANES 2005–2010) data. By focusing on circulating vitamin B_6_ metabolites rather than dietary intake alone, we sought to complement and extend previous work by (1) jointly examining PLP and PA, (2) exploring potential dose–response relationships and (3) assessing effect modification across key demographic and clinical subgroups through stratified analyses.

## Materials and methods

### Study population

**Figure 1.**
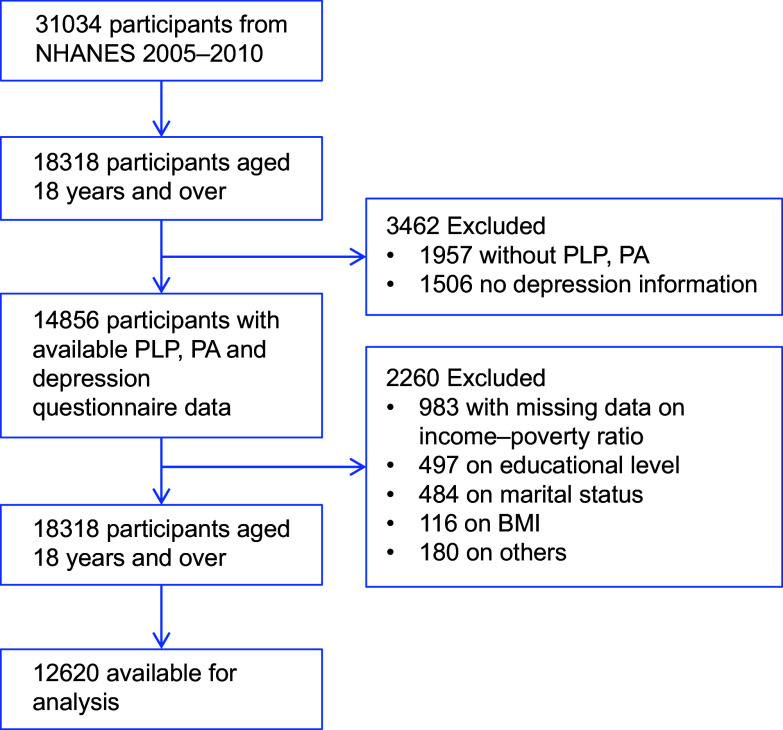
Flow chart of the population included in our study.

NHANES constitutes a comprehensive, nationally representative surveillance initiative aimed at assessing the health and nutritional status, both physical and psychological, of the non-institutionalised civilian population within the USA. This programme, operating under the ethical oversight of the National Center for Health Statistics Institutional Review Board, annually samples approximately 5000 individuals to ensure national representativeness. Data collection encompasses demographic, nutritional, laboratory and questionnaire-based information. Participants provide informed consent, and survey data are disseminated biennially basis^(17–19)^. PLP and PA measurements were available through the 2009–2010 cycle, with consistent detection methodology employed from 2005 to 2010. For this analysis, we initially identified 31 034 participants from three consecutive cycles (NHANES 2005–2006, 2007–2008 and 2009–2010) to examine the association between vitamin B_6_ metabolites and depression. After excluding participants lacking PLP or PA measurements, Patient Health Questionnaire-9 (PHQ-9) data, those under 18 years of age and those with missing covariate information, the final analytical sample comprised 12 620 participants (Figure 1).

### Depression assessment

Depression symptoms were evaluated using the PHQ-9, a validated self-report instrument comprising nine items based on depression diagnostic criteria from the American Psychiatric Association’s Diagnostic and Statistical Manual-IV^(20)^. Each PHQ-9 item utilises a four-point Likert scale ranging from 0 (‘not at all’) to 3 (‘nearly every day’), yielding a total score between 0 and 27. A PHQ-9 score ≥10 was designated as indicative of depression, consistent with established thresholds demonstrating 74 % sensitivity and 91 % specificity in primary care settings^(21)^.

### Measurement of vitamin B_6_ status

Vitamin B_6_ status was assessed using serum PLP, the biologically active coenzyme form and PA, the principal catabolic and urinary excretion product of vitamin B_6_ (in nmol/L). Both biomarkers were quantified using HPLC.^(10,22)^ Comprehensive quality control procedures and detailed analytical protocols are documented in the Mobile Examination Center laboratory procedure manual available on the NHANES website^(23)^.

### Covariates

Based on established literature and clinical relevance, we incorporated multiple covariates including age, gender, race, marital status, education level, poverty–income ratio, physical activity, BMI, estimated glomerular filtration rate (eGFR), alcohol consumption, smoking status and history of diabetes, hypertension and liver disease^(24,25)^. Racial/ethnic categories comprised non-Hispanic White, non-Hispanic Black, other Hispanic, Mexican American and other racial groups. Marital status was classified as partnered (married) or unpartnered (unmarried). Educational attainment was classified as less than high school completion *v*. high school graduation or higher. Poverty–income ratio was stratified into three tiers: <1·3, 1·3–3·49 and >3·5, with higher values indicating greater household income relative to the federal poverty threshold^(26)^. Physical activity was defined as engagement in moderate or vigorous intensity exercise for ≥10 consecutive minutes outside of occupational or transportation contexts, while physical inactivity denoted <10 min of such activities^(27)^. BMI was calculated as weight (kg) divided by height squared (m^2^). eGFR was derived using the Chronic Kidney Disease Epidemiology Collaboration creatinine equation^(28)^. Smoking status was classified as non-smokers (<100 lifetime cigarettes) or smokers (≥100 lifetime cigarettes). Alcohol consumption was categorised as non-drinkers (<12 alcoholic beverages in the preceding year) or drinkers (≥12 alcoholic beverages in the preceding year). Diabetes was defined by self-reported physician diagnosis or current insulin use. Hypertension was determined by self-reported physician diagnosis or blood pressure measurements exceeding 140/90 mmHg. Liver disease history was ascertained through participant interviews.

### Statistical analysis

This study adhered to STROBE guidelines. Statistical analyses were performed using R software (version 4.4.1) with statistical significance defined as a *P* value <0·05. Continuous variables were presented as mean ± s d or median with interquartile range (IQR), while categorical variables were expressed as frequencies and percentages. Specifically, vitamin B_6_ markers (PLP and PA) were presented as median (IQR) due to their skewed distributions, while other continuous variables (e.g., age and BMI) were presented as mean ± s d given their approximately normal distributions. Serum PLP and PA concentrations were categorised into quartiles, with the lowest quartile (Q1) serving as the reference group. The associations between serum vitamin B_6_ metabolites and depression risk were examined using multivariable logistic regression models, yielding OR with corresponding 95 % CI. Two sequential adjustment models were constructed: model 1 adjusted solely for age and gender, while model 2 for serum PLP incorporated additional adjustments for race, marital status, education level, poverty–income ratio, physical activity, BMI, alcohol consumption, smoking status and history of diabetes, hypertension and liver disease. For serum PA analyses, model 2 included all aforementioned covariates plus estimated glomerular filtration rate. We first calculated correlation coefficients to quantify the association between serum PLP and PA. To further examine whether PLP and PA contribute independently to depression risk, we conducted sensitivity analyses in which both metabolites were entered simultaneously as continuous predictors in the same multivariable logistic regression model. Potential non-linear dose–response relationships between PLP, PA and depression were explored using restricted cubic spline functions with four knots placed at the 5th, 35th, 65th and 95th percentiles of the metabolite distributions, with the median value used as the reference point. Non-linearity was assessed using likelihood ratio tests comparing models with and without the spline terms.

## Results

### Baseline characteristics of the study population

Of the 12 620 participants included in the analysis, 1070 (8·5 %) were classified as having depression based on PHQ-9 scores ≥10, while 11 550 (91·5 %) were classified as non-depressed. Individuals with depression exhibited lower serum concentrations of both vitamin B_6_ metabolites compared with their non-depressed counterparts. Specifically, median serum PLP in the depression group was 31·7 nmol/L (IQR: 20·3–55·85 nmol/L) *v*. 44·6 nmol/L (IQR: 26·8–77·8 nmol/L) in the non-depression group. Similarly, median serum PA was 20·3 nmol/L (IQR: 13·3–34·8 nmol/L) among depressed participants *v*. 25·4 nmol/L (IQR: 16·0–51·2 nmol/L) among non-depressed participants. Significant between-group differences were observed across multiple demographic and clinical characteristics, including age, gender, race, marital status, education level, poverty–income ratio, BMI, estimated glomerular filtration rate, smoking status and history of diabetes, hypertension and liver disease. Detailed baseline characteristics stratified by depression status are presented in Table 1.

**Table 1.** Baseline characteristics of included participants (NHANES 2005–2010)

Characteristic	Total (*n* 12 620)		Without depression (*n* 11 550)		With depression (*n* 1070)		*P*
	*n*	%	*n*	%	*n*	%	
Age(year)							<0·001
Mean	49·12		49·33		46·91		
s d	17·86		18·06		15·44		
Gender							<0·001
Male	6206	49·2	5824	50·4	382	35·7	
Female	6414	50·8	5726	49·6	688	64·3	
Race							<0·001
Non-Hispanic White	6468	51·3	5978	51·8	490	45·8	
Non-Hispanic Black	2424	19·2	2192	19·0	232	21·7	
Other Hispanic	990	7·8	873	7·6	117	10·9	
Mexican American	2237	17·7	2046	17·7	191	17·9	
Other	501	4·0	461	4·0	40	3·7	
Marital status							<0·001
Unmarried	4791	38·0	4247	36·8	544	50·8	
Married	7829	62·0	7303	63·2	526	49·2	
Educational level							<0·001
Below high school	3428	27·2	3014	26·1	414	38·7	
High School or above	9192	72·8	8536	73·9	656	61·3	
Income–poverty ratio							<0·001
<1·3	3696	29·3	3132	27·1	564	52·7	
1·3–3·49	4829	38·3	4481	38·8	348	32·5	
>3·49	4095	32·4	3937	34·1	158	14·8	
Physical activity							<0·001
Inactive	6181	49·0	5425	47·0	756	70·7	
Active	6439	51·0	6125	53·0	314	29·3	
BMI (kg/m^2^)	29·07	6·76	28·93	6·60	30·55	8·09	<0·001
Mean	98·22		97·87		102·04		
s d	24·49		24·50		24·03		
eGFR (ml/min/1·73m^2^)							<0·001
Mean	98·22		97·87		102·04		
s d	24·49		24·50		24·03		
PLP (nmol/L)							<0·001
Median	43·40		44·60		31·70		
IQR	26·00, 77·03		26·80, 78·80		20·30, 55·85		
PA (nmol/L)							<0·001
Median	24·80		25·40		20·30		
IQR	15·70, 49·42		16·00, 51·18		13·30, 34·77		
Smoking status							
No	6590	52·2	6166	53·4	424	39·6	<0·001
Yes	6030	47·8	5384	46·6	646	60·4	
Alcohol consumption	98·22	24·49					0·419
No	3575	28·3	3260	28·2	315	29·4	
Yes	9045	71·7	8290	71·8	755	70·6	
Diabetes							<0·001
No	11 167	88·5	10 284	89·0	883	82·5	
Yes	1453	11·5	1266	11·0	187	17·5	
Hypertension							<0·001
No	7747	61·4	7176	62·1	571	53·4	
Yes	4873	38·6	4374	37·9	499	46·6	
Liver disease							<0·001
No	12 209	96·7	11 212	97·1	997	93·2	
Yes	411	3·3	338	2·9	73	6·8	

eGFR, estimated glomerular filtration rate; PLP, pyridoxal 5′-phosphate; IQR, interquartile range; PA, 4-pyridoxal acid.

### Association between serum vitamin B_6_ metabolites and depression

To elucidate the association between vitamin B_6_ metabolites and depression, we performed multivariable logistic regression analyses with serum PLP and PA categorised into quartiles. When examining serum PLP, participants in higher quartiles exhibited progressively lower odds of depression compared with those in the lowest quartile (Q1, <26·00 nmol/L). Specifically, the fully adjusted odds ratios for depression were 0·79 (95 % CI: 0·66–0·93, *P* < 0·001) for Q2 (26·00–43·40 nmol/L), 0·67 (95 % CI: 0·55–0·81, *P* < 0·001) for Q3 (43·40–77·03 nmol/L) and 0·70 (95 % CI: 0·57–0·85, *P* < 0·001) for Q4 (≥77·03 nmol/L), with a significant dose-response relationship (*P*-trend <0·001). This inverse association persisted across all adjustment models (Table 2). Similarly, serum PA demonstrated an inverse association with depression, though with attenuated statistical significance in middle quartiles. In the fully adjusted model (model 2), compared with participants in the lowest quartile (Q1 <15·70 nmol/L), those in Q2 (15·70–24·80 nmol/L), Q3 (24·80–49·42 nmol/L) and Q4 (≥49·42 nmol/L) demonstrated odds ratios of 0·94 (95 % CI: 0·79, 1·12, *P* = 0·47), 0·86 (95 % CI: 0·71, 1·04, *P* = 0·12) and 0·80 (95 % CI: 0·65, 0·98, *P* < 0·05), respectively, with a significant trend across quartiles (*P*-trend <0·001) (Table 3).

**Table 2.** Association between serum PLP and depression

Serum PLP (nmol/L)	Non-adjusted model			Model 1			Model 2		
	OR	95 % CI	*P*	OR	95 % CI	*P*	OR	95 % CI	*P*
Quartiles									
Q1 (<26·00)	1	Ref		1	Ref		1	Ref	
Q2 (26·00–43·40)	0·64	0·54–0·75	<0·001	0·65	0·55–0·76	<0·001	0·79	0·66–0·93	<0·001
Q3 (43·40–77·03)	0·44	0·37–0·52	<0·001	0·47	0·39–0·56	<0·001	0·67	0·55–0·81	<0·001
Q4 (≥77·03)	0·39	0·32–0·46	<0·001	0·42	0·34–0·50	<0·001	0·70	0·57–0·85	<0·001
Trend test			<0·001			<0·001			<0·001

PLP, pyridoxal 5′-phosphate.

Model 1 was adjusted for age and gender. Model 2 was adjusted for age, gender, race, marital status, education level, income–poverty ratio, physical activity, BMI, smoking status, alcohol consumption, diabetes, hypertension and liver disease. *P*_for trend_ was calculated by assigning the median value to each quartile and treating it as a continuous variable in the logistic regression model.

**Table 3.** Association between serum PA and depression

Serum PA (nmol/L)	Non-adjusted model			Model 1			Model 2		
	OR	95 % CI	*P*	OR	95 % CI	*P*	OR	95 % CI	*P*
Quartiles									
Q1 (<15·70)	1	Ref		1	Ref		1	Ref	
Q2 (15·70–24·80)	0·76	0·64–0·90	<0·01	0·82	0·69–0·97	<0·05	0·94	0·79–1·12	0·47
Q3 (24·80–49·42)	0·60	0·50–0·71	<0·001	0·67	0·56–0·80	<0·001	0·86	0·71–1·04	0·12
Q4 (≥49·42)	0·47	0·39–0·57	<0·001	0·54	0·44–0·65	<0·001	0·80	0·65–0·98	<0·05
Trend test			<0·001			<0·001			<0·001

PA, 4-pyridoxic acid.

Model 1 was adjusted for age and gender. Model 2 was adjusted for age, gender, race, marital status, education level, income–poverty ratio, physical activity, BMI, eGFR,smoking status, alcohol consumption, diabetes, hypertension and liver disease. *P*_for trend_ was calculated by assigning the median value to each quartile and treating it as a continuous variable in the logistic regression model.

### Dose–response relationship

**Figure 2.**
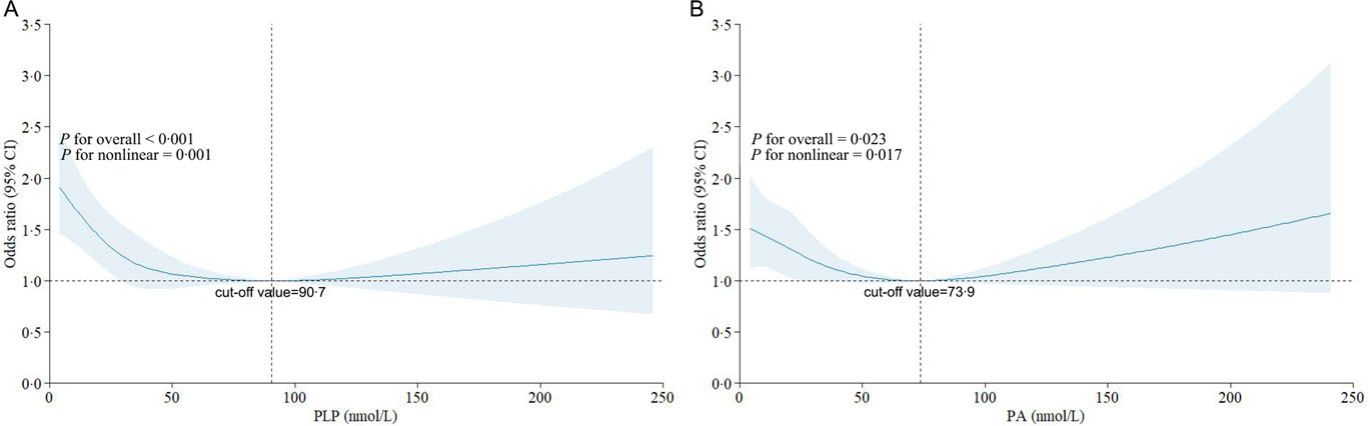
Association between serum PLP, PA and depression using a restricted cubic spline regression model. Figure 2A shows OR for depression according to serum PLP adjusted for age, gender, race, marital status, education level, income–poverty ratio, physical activity, BMI, alcohol consumption, smoking status, diabetes, hypertension and liver disease. Figure 2B shows OR for status according to PA adjusted for age, gender, race, marital status, education level, income–poverty ratio, physical activity, BMI, alcohol consumption, smoking status, diabetes, hypertension, liver disease and eGFR. Data were fitted by a logistic regression model, and the model was conducted with four knots at the 5th, 35th, 65th and 95th percentiles of serum PLP. Solid lines indicate OR, and shadow shape indicates 95 % CI. PA, 4-pyridoxic acid; PLP, pyridoxal 5′-phosphate.

To assess potential non-linear relationships between vitamin B_6_ metabolites and depression, we employed restricted cubic spline modelling. The analysis revealed a significant non-linear association between serum PLP and depression risk (*P*_for non-linearity_ = 0·001), characterised by a reversed L-shaped pattern. We identified an inflection point at 90·6 nmol/L that demarcated distinct relationships. At concentrations below this threshold, serum PLP demonstrated a significant inverse association with depression (OR = 0·992, 95 % CI: 0·988–0·996, *P* < 0·001), whereas no significant association was detected above this threshold (*P* = 0·353) (Figure 2a).

For serum PA, we also observed a significant non-linear dose–response relationship with depression risk (*P*_for non-linearity_ = 0·017), but with a distinct U-shaped pattern and an inflection point at 73·9 nmol/L. Below this concentration, serum PA exhibited an inverse association with depression (OR = 0·994, 95 % CI: 0·988–0·999, *P* < 0·05), analogous to the protective effect observed with PLP. However, at concentrations exceeding 73·9 nmol/L, serum PA demonstrated a positive association with depression risk (OR = 1·008, 95 % CI: 1·002–1·013, *P* < 0·01) (Figure 2b). Given that PA is the terminal excretion product of vitamin B_6_, these elevated concentrations are more likely to indicate a state of accelerated vitamin B_6_ turnover than a direct harmful effect of PA itself.

Because only a relatively small proportion of participants had metabolite concentrations above the spline-derived inflection points, particularly for PA, the majority of individuals in the top quartiles fell within the concentration range where the associations remained inverse. In our data, approximately 5 % of participants had PA levels above 73·9 nmol/L (data not shown). Consequently, the quartile-based logistic regression analyses primarily capture the overall decreasing risk pattern across the concentration range that includes most participants, whereas the RCS models, which exploit the full continuous information, are more sensitive to detecting the upturn in depression risk at the extreme upper tail of the PA distribution.

### Subgroup analyses

**Figure 3.**
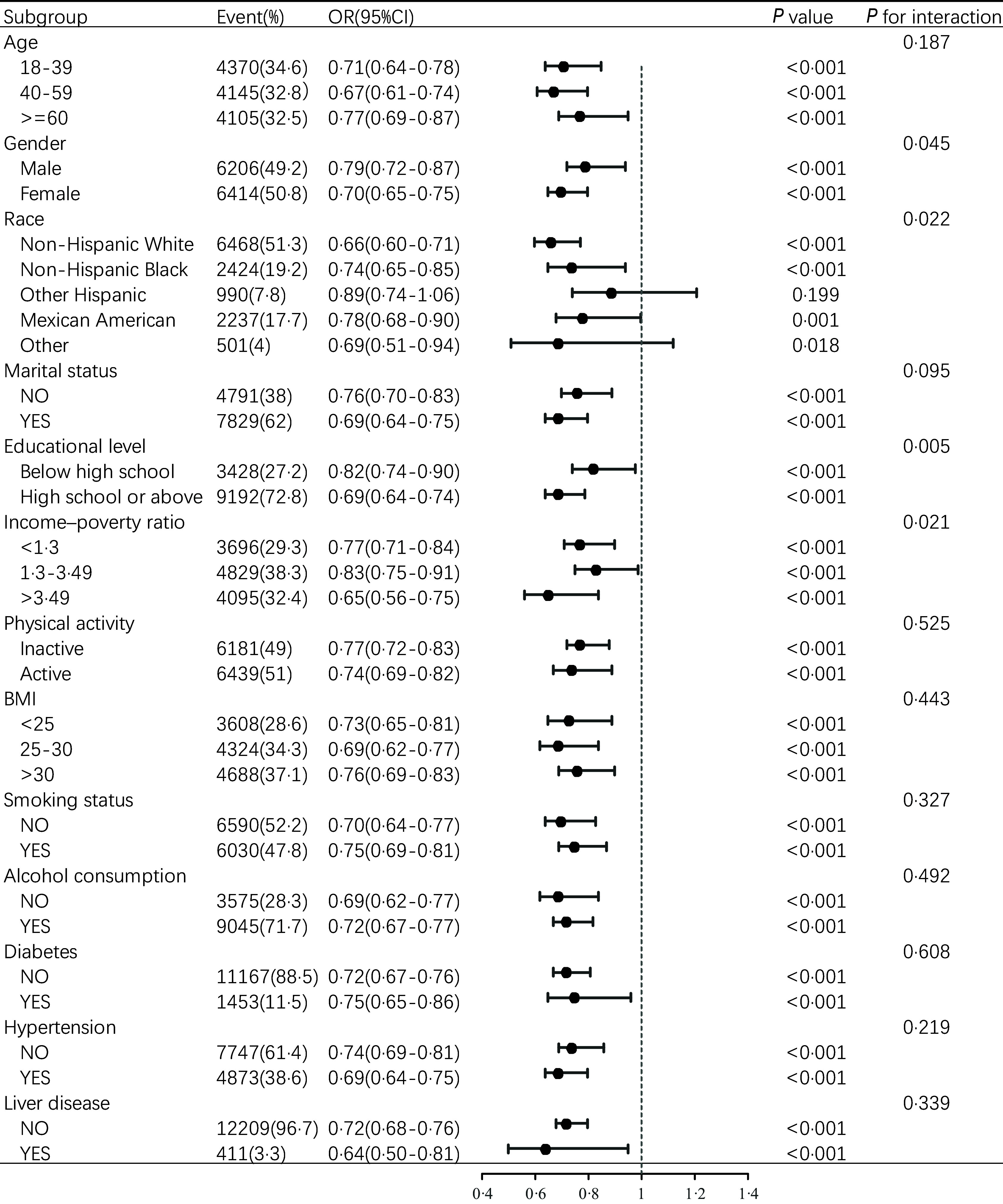
Figure 3 long description. Stratified analyses of the association between serum PLP and depression. Except for the stratification component itself, the stratifications were adjusted for age, gender, race, marital status, education level, income–poverty ratio, physical activity, BMI, alcohol consumption, smoking status, diabetes, hypertension and liver disease. PLP, pyridoxal 5′-phosphate.

**Figure 4.**
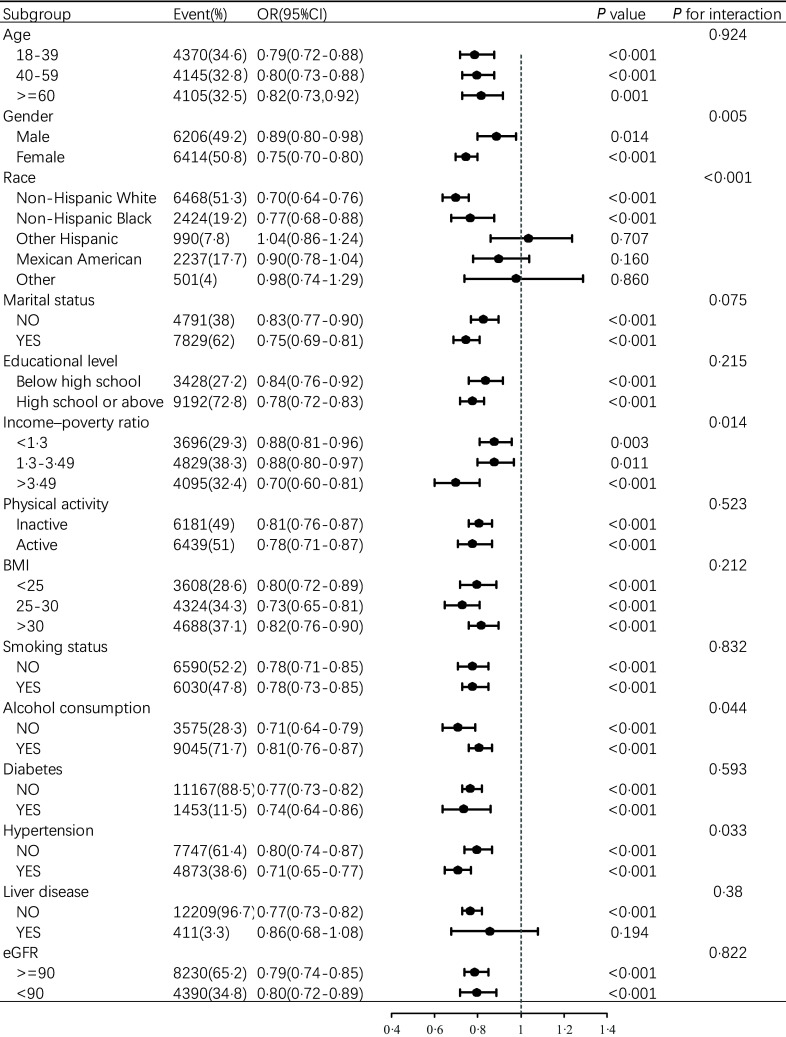
Stratified analyses of the association between serum PA and depression. Except for the stratification component itself, the stratifications were adjusted for age, gender, race, marital status, education level, income–poverty ratio, physical activity, BMI, alcohol consumption, smoking status, diabetes, hypertension, liver disease and eGFR. eGFR, estimated glomerular filtration rate; PA, 4-pyridoxic acid.

We conducted subgroup analyses to examine potential heterogeneity and effect modification. The inverse associations between both serum PLP and PA with depression remained directionally consistent across most subgroups examined. However, these associations failed to reach statistical significance in specific populations, including other Hispanic and other racial categories for both metabolites and among individuals with liver disease for PA. The protective association between serum PLP and depression was particularly robust among females, Non-Hispanic Whites, married individuals and those with poverty–income ratios exceeding 3·49 (Figure 3). Similarly, the inverse relationship between serum PA and depression was most pronounced within these same demographic subgroups (Figure 4). Interaction analyses revealed significant effect modification of the PLP–depression relationship by gender, race, educational level and poverty–income ratio (all *P*_for interaction_ < 0·05). The association between serum PA and depression was significantly modified by gender, race, poverty–income ratio, alcohol consumption and hypertension status (all *P*_for interaction_ < 0·05). It is noteworthy, however, that these associations were not statistically significant in all subgroups, such as among individuals of ‘Other Race’. These non-significant findings should be interpreted with caution, as they likely stem from limited statistical power due to smaller sample sizes within these specific demographics.

### Correlation between pyridoxal 5′-phosphate and 4-pyridoxic acid and mutually adjusted models

Serum PLP and PA were moderately positively correlated (Spearman correlation coefficient *r* 0·582, *P* < 0·001; Supplementary Figure 1). In sensitivity analyses including both PLP and PA as continuous predictors in the same multivariable logistic regression model, the inverse association between serum PLP and depression remained statistically significant (OR = 0·998, 95 % CI: 0·997–0·999, *P* = 0·002), whereas the association for serum PA was attenuated and became non-significant (OR = 0·999, 95 % CI: 0·999–1·001, *P* = 0·58; Supplementary Table 1).

## Discussion

Our study revealed nonlinear inverse associations between serum vitamin B_6_ metabolites and depression, with distinct threshold effects for both PLP and PA. These findings are broadly consistent with, and extend, those of a recent NHANES analysis by Lu et al.^(15)^ who reported that higher dietary vitamin B_6_ intake and higher plasma PLP quartiles were associated with a lower prevalence of depression in USA adults. Similar to their results, we observed a monotonic decrease in depression odds across PLP quartiles. By additionally modelling continuous PLP and PA concentrations using restricted cubic splines, and by incorporating PA as a second circulating biomarker rather than dietary intake, we further demonstrate that the PLP–depression association follows an L-shaped pattern with a threshold around 90·6 nmol/L, whereas PA exhibits a U-shaped relationship with depression risk. The mutual adjustment analysis indicates that the association of PLP with depression is independent of PA, while that of PA is not independent of PLP. Moreover, in contrast to previous studies^(14,29–34)^, our subgroup analyses identify important effect modifications by age, sex and socio-economic characteristics, thereby providing a more detailed picture of vitamin B_6_ metabolism and depression risk in a demographically diverse adult population. These extensions suggest that the vitamin B_6_–depression relationship is not only present, as suggested by other prior studies, but also metabolite specific and threshold dependent. Taken together, the quartile-based logistic regression and spline analyses provide complementary information: the quartile models summarise the overall inverse association across the concentration range that encompasses the vast majority of participants, whereas the spline models highlight that a small subset of individuals with very high PA levels deviates from this pattern, giving rise to the observed U-shaped relationship.

Several plausible mechanisms may explain the relationship between vitamin B_6_ status and depression. Vitamin B_6_ derives from both dietary sources and gut microbiota production, followed by intestinal absorption and circulates predominantly as PLP, which serves as an essential cofactor for DOPA decarboxylase and glutamate decarboxylase, enzymes crucial for the biosynthesis of serotonin, dopamine and GABA, respectively^(35,36)^. Serotonin plays a central role in mood regulation, and its deficiency has long been implicated in depression pathophysiology^(37)^, while imbalances in dopaminergic and GABAergic neurotransmission have also been associated with depressive symptoms and related affective disturbances^(38–40)^. Classical biochemical studies have shown that PLP availability regulates the holoenzyme content and activity of key PLP-dependent decarboxylases in brain tissue, including aromatic L-amino acid decarboxylase and glutamate decarboxylase^(41,42)^. Experimental work in rodents further demonstrates that pyridoxine deficiency lowers seizure threshold and alters GABAergic signalling, whereas pharmacological vitamin B_6_ supplementation exerts anxiolytic- and antidepressant-like effects, in part through GABAergic and NO–sGC–cGMP pathways^(43,44)^. In non-human primates, positron emission tomography studies have indicated that pyridoxine administration can increase the rate of serotonin synthesis in the brain^(45)^. Together, these findings support a biologically plausible role for vitamin B_6_-dependent neurotransmitter pathways in mood regulation and are consistent with the inverse associations between vitamin B_6_ status and depression observed in our study.

Research has demonstrated a significant positive correlation between PLP and PA concentrations in both cerebrospinal fluid and plasma^(46)^, suggesting that peripheral vitamin B_6_ markers may reflect central nervous system status. This relationship is further supported by animal studies showing that vitamin B_6_ deficiency reduces PLP and dopamine concentrations in the prefrontal cortex, accompanied by decreased social behaviour^(47)^. Therefore, the lower serum PLP and PA levels observed in depressed individuals in our study may reflect similar reductions in the central nervous system, potentially compromising neurotransmitter synthesis involved in depression. Additionally, vitamin B_6_ exhibits antioxidant properties in brain tissue, with supplementation shown to decrease oxidative stress-induced memory dysfunction^(48)^ and enhance neuronal resilience to oxidative neurotoxicity by promoting glutathione synthesis^(49)^. Given that oxidative stress represents a key pathogenic factor in depression^(50,51)^, this antioxidant function may provide another mechanism linking vitamin B_6_ status to depression risk. Finally, the relationship may be bidirectional, as depressed individuals often experience reduced appetite, potentially further decreasing vitamin B_6_ intake^(52)^.

An interesting aspect of our findings is the different non-linear patterns observed for PLP and PA. PLP is the main coenzyme form of vitamin B_6_ and directly participates in neurotransmitter synthesis, one-carbon metabolism and other pathways implicated in depression^(53)^. In contrast, PA is the terminal oxidation and major urinary excretion product of vitamin B_6_ and has no known coenzyme or signaling roles; it is best viewed as an indicator of vitamin B_6_ catabolism and turnover rather than an active metabolite. Within this framework, the positive association between very high PA concentrations and depression risk in our spline models is unlikely to reflect a direct toxic or ‘adverse’ effect of PA or pyridoxine itself. Rather, elevated PA may mark states of increased vitamin B_6_ utilisation and breakdown, for example under conditions of inflammation or oxidative stress that accompany depression^(13)^. The finding that the association between PLP and depression remained significant after adjusting for PA, while the association for PA was attenuated and became non-significant when adjusted for PLP, suggests that the link between vitamin B_6_ status and depression is more directly reflected by the level of the biologically active coenzyme, PLP. The moderate positive correlation between PLP and PA (*r* 0·582) indicates that PA levels are, to a considerable extent, a function of PLP availability and its subsequent catabolism. Therefore, the apparent U-shaped association between PA and depression observed in the spline model, which did not persist after accounting for PLP, may primarily reflect the complex interplay of vitamin B_6_ metabolism and turnover states, rather than an independent effect of PA itself. This reinforces the interpretation that PA serves best as a marker of vitamin B_6_ catabolism and that PLP is the central metabolite of interest in the context of depression. Over time, sustained high turnover could deplete total vitamin B_6_ pools, leading to low levels of both PLP and PA, which is consistent with our observation that participants in the lowest PA quartile also had elevated odds of depression. Importantly, neuropathy due to pyridoxine toxicity typically requires prolonged exposure to pharmacological doses (around 200 mg/day or higher)^(54)^ and is considered rare in the general population; thus, it is unlikely that overt pyridoxine toxicity explains the elevated PA levels at the upper end of our distribution. Our cross-sectional data cannot determine temporal ordering, and any dynamic trajectory of PA across different stages or durations of depressive illness should therefore be regarded as speculative and hypothesis-generating, to be tested in future longitudinal studies.

Our findings carry notable implications for both clinical practice and public health. The inflection point for the PLP–depression association was identified at approximately 90·7 nmol/L, a concentration substantially higher than the conventional biochemical deficiency cut-off of 20 nmol/L^(55)^. This discrepancy suggests that vitamin B_6_ levels deemed ‘adequate’ by traditional nutritional standards might be suboptimal for mental health, pointing to a potential need to re-evaluate the optimal range for neurological and psychiatric well-being^(56)^. From a public health perspective, ensuring an optimal vitamin B_6_ status could thus be a valuable component of population-level strategies for the primary prevention of depression. This could be achieved through promoting a diversified diet rich in natural B_6_ sources (e.g., fish, poultry, chickpeas and potatoes) or, where dietary intake is insufficient, considering prudent food fortification or supplementation strategies^(57,58)^. Future longitudinal studies and randomised controlled trials are needed to confirm a causal relationship and to define precise, mentally optimal target ranges for serum vitamin B_6_ metabolites.

In this study, we examined the associations between serum vitamin B_6_ metabolites (PLP and PA) and depression prevalence among USA adults. Our investigation offers several methodological strengths. First, we utilised data from NHANES, a nationally representative database with rigorous quality control procedures, enhancing the validity and generalisability of our findings. Second, we assessed vitamin B_6_ status using serum biomarkers rather than dietary intake estimates. This approach provides a more accurate reflection of biologically available vitamin B_6_, accounting for both dietary sources and gut microbiota contributions while avoiding recall biases inherent in dietary interviews. Furthermore, these metabolites were measured using LC/MS/MS methodology, the current gold standard for serum vitamin B_6_ quantification. Third, we employed restricted cubic spline regression to characterise the potentially nonlinear dose–response relationships between serum vitamin B_6_ metabolites and depression risk.

### Limitations

Despite these strengths, our study has important limitations. The cross-sectional design precludes causal inference regarding the protective effect of vitamin B_6_ against depression. Prospective studies are needed to establish temporal relationships and potential causality. Additionally, although we adjusted for numerous potential confounders, residual confounding from unmeasured or unknown factors cannot be ruled out.

### Conclusion

In conclusion, our study demonstrates nonlinear inverse associations between serum vitamin B_6_ metabolites and depression in USA adults, with particularly pronounced relationships among females, Non-Hispanic Whites and individuals with higher income levels. These findings suggest that maintaining adequate – but not excessive – serum vitamin B_6_ status is associated with a lower prevalence of depression. The distinct nonlinear patterns observed for PLP (L-shaped) and PA (U-shaped) indicate the complex relationship between vitamin B_6_ metabolism and mental health. However, large-scale prospective studies are needed to establish temporal relationships and causality, while large-scale prospective studies are needed to establish temporal relationships and causality, and further mechanistic work, building on existing biochemical and experimental evidence, is required to clarify how specific vitamin B_6_-dependent pathways influence depression pathophysiology in humans.

## Supporting information

10.1017/S0007114526106321.sm001 Mai et al. supplementary material 1 Mai et al. supplementary material

10.1017/S0007114526106321.sm002 Mai et al. supplementary material 2 Mai et al. supplementary material

## Supplementary material

To view supplementary material for this article, please visit https://doi.org/10.1017/S0007114526106321

## Figure 3 Long description

The table presents stratified analyses of the association between serum PLP and depression, adjusted for multiple factors. It includes 15 rows and 6 columns. The columns are labeled Subgroup, Event(%), OR(95%CI), P value, and P for interaction. The subgroups include Age, Gender, Race, Marital status, Educational level, Income-poverty ratio, Physical activity, BMI, Smoking status, Alcohol consumption, Diabetes, Hypertension, and Liver disease. Each subgroup is further divided into specific categories with corresponding Event(%), OR(95%CI), P value, and P for interaction values. For example, under Age, the categories are 18-39, 40-59, and >=60, with Event(%) values of 34.6, 32.8, and 32.5 respectively. The table provides detailed data on the odds ratios and p-values for each category, indicating the strength and significance of the association between serum PLP and depression within each subgroup.

Navigate back to Figure 3.
