# Supplementary figures and images for: Association between serum vitamin B6 levels and depression in adults: a cross-sectional National Health and Nutrition Examination Survey (NHANES) study
Source: Br J Nutr. 2026 Feb 2;135(9):995–1005. doi: 10.1017/S0007114526106321 (PMC13315548; doi:10.1017/S0007114526106321)

**$r = 0.582$   $P = <2e-16$**

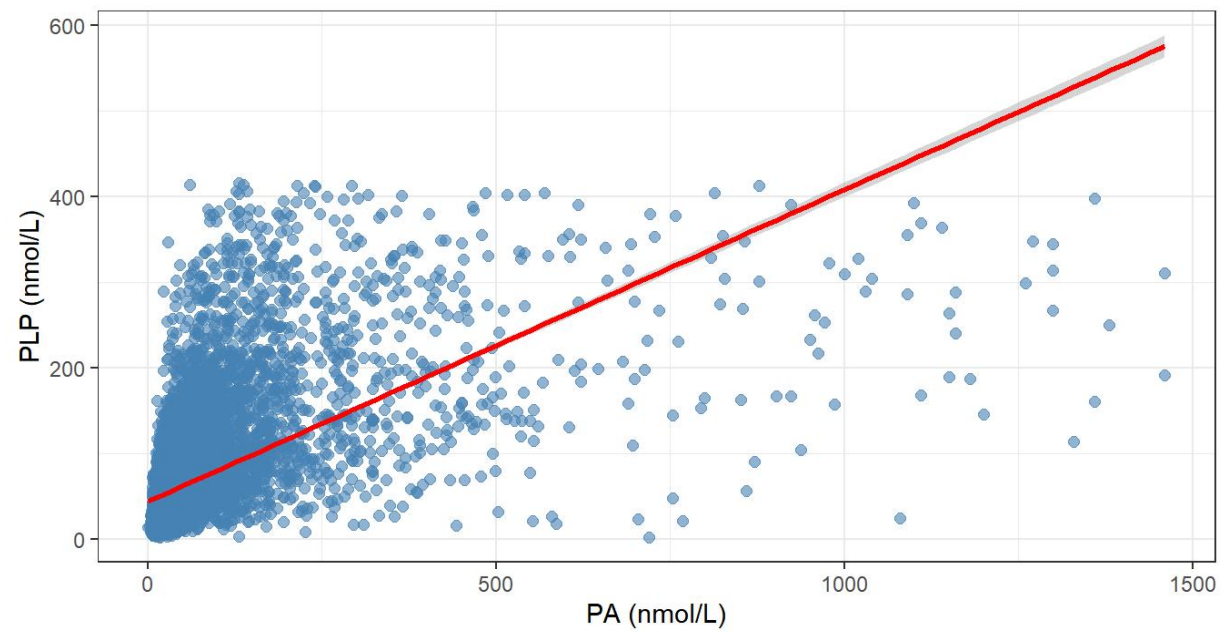

Supplement: Mai et al. supplementary material 1 — Mai et al. supplementary material [file S0007114526106321sup001.pdf]
